# *In Vitro* Analysis of Predicted DNA-Binding Sites for the Stl Repressor of the *Staphylococcus aureus* SaPIBov1 Pathogenicity Island

**DOI:** 10.1371/journal.pone.0158793

**Published:** 2016-07-07

**Authors:** Veronika Papp-Kádár, Judit Eszter Szabó, Kinga Nyíri, Beata G. Vertessy

**Affiliations:** 1 Department of Applied Biotechnology and Food Science, Budapest University of Technology and Economics, Budapest, 1111, Hungary; 2 Laboratory of Genome Metabolism and Repair, Institute of Enzymology, Research Centre for Natural Sciences, Hungarian Academy of Sciences, Budapest, 1117, Hungary; University of Manchester, UNITED KINGDOM

## Abstract

The regulation model of the *Staphylococcus aureus* pathogenicity island SaPIbov1 transfer was recently reported. The repressor protein Stl obstructs the expression of SaPI proteins Str and Xis, latter which is responsible for mobilization initiation. Upon Φ11 phage infection of *S*. *aureus*. phage dUTPase activates the SaPI transfer via Stl-dUTPase complex formation. Our aim was to predict the binding sites for the Stl repressor within the *S*. *aureus* pathogenicity island DNA sequence. We found that Stl was capable to bind to three 23-mer oligonucleotides, two of those constituting sequence segments in the *stl-str*, while the other corresponding to sequence segment within the *str-xis* intergenic region. Within these oligonucleotides, mutational analysis revealed that the predicted binding site for the Stl protein exists as a palindromic segment in both intergenic locations. The palindromes are built as 6-mer repeat sequences involved in Stl binding. The 6-mer repeats are separated by a 5 oligonucleotides long, nonspecific sequence. Future examination of the interaction between Stl and its binding sites *in vivo* will provide a molecular explanation for the mechanisms of gene repression and gene activation exerted simultaneously by the Stl protein in regulating transfer of the SaPIbov1 pathogenicity island in *S*. *aureus*.

## Introduction

*Staphylococcus aureus* pathogenicity islands (SaPIs) have a major role in spreading virulence genes among bacterial populations [1], as they encode major antigens responsible for different toxinoses, such as the toxin of Toxic Shock Syndrome (TSS). The SaPI life cycle can be initiated and realized only in the presence of certain helper bacteriophages, which counteract the SaPI repressor (Stl) and provide the proteins necessary for SaPI packaging into infectious particles [2–4]. As a result of helper phage infection or prophage activation, the repression is released by a de-repressor protein which interacts with the repressor Stl. It was shown that in case of SaPIbov1 and SaPIbov5 pathogenicity islands, dUTPases of Φ11 and 80α helper phages, respectively, act as de-repressor proteins [3]. De-repression leads to the expression of SaPI excision proteins. When SaPI becomes de-repressed and mobilized, the SaPI DNA is replicated and then it is packaged into transducing phage particles formed by helper phage encoded structural proteins [1,5]. It is compelling, that the de-repressor proteins of Φ11 and 80α helper phages, namely the respective dUTPase proteins also have well-known role in the nucleotide metabolism [6].

dUTPases are responsible for hydrolyzing dUTP and essential in maintaining DNA integrity by controlling relative cellular levels of dTTP/dUTP, both of which can be incorporated into newly synthesized DNA [7–9]. Uracil-excision DNA repair is responsible for the removal of uracil, but high levels of uracil in DNA trigger double-strand breaks and lead to cell death [8,10–13]. dUTPases are widely known to be ubiquitous proteins [14,15], and are encoded by several viruses, including bacteriophages [16,17]. Interestingly, in *S*. *aureus*, the bacterial genome does not encode this important protein [18–21]. However, the dUTPase gene is carried frequently (and may be potentially expressed) by integrated prophages [21]. It was recently shown that Φ11 phage dUTPase and Stl_SaPIbov1_ (referred to as Stl in the present study) form a strong complex [3,21]. It was shown that Stl is a competitive, slow and tight binding inhibitor of Φ11 dUTPase. Based on these data, a model was proposed for the dUTPase-regulated molecular switch [21].

Although the mechanism of the de-repression was investigated at the molecular level, the mechanism of binding of Stl to its DNA target is not understood. The expression pattern of SaPIbov1 genes that may be regulated by Stl was investigated in two studies including the β-lactamase and LacZ reporter systems, respectively [2,22]. In the first article [2], it was proposed that Stl represses the expression of *str* (a potential transcriptional regulator), *xis* (excisionase), and *rep* (replicon specific initiator) genes. It was also suggested that Stl promotes its own expression [2]. This scenario is in agreement with the fact that Stl is the master repressor of SaPI reproduction, which starts with the excision of the SaPI [1]. However, the experimental setup was designed in such a way that either the full-length SaPIbov1 segment was present in the system, or the gene for Stl was deleted from this segment and comparative expression of the different proteins was then investigated. In this system in the absence of Stl, the expression of other transcriptional regulators, e.g., Str, may have increased and may have contributed to the overall expression pattern. Hence the obtained results in this experimental setup may be somewhat biased and should be treated with caution. The second study, in which the test system was SaPIbov1 independent, reinforced that Stl inhibits the expression of the *xis* gene, and showed that it represses also the *int* (integrase) gene [22].

**Fig 1:**
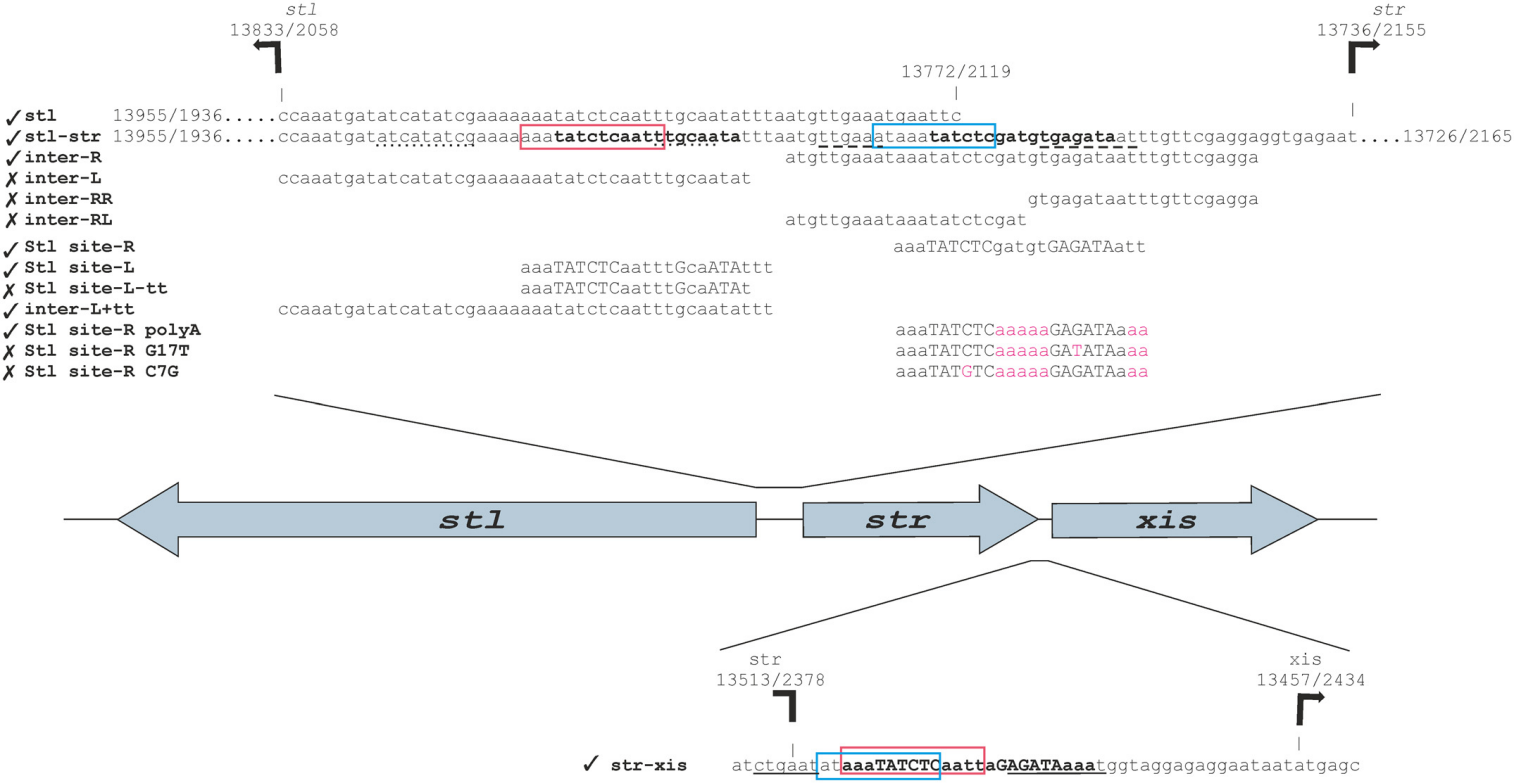
Rational design of oligonucleotides for the identification of Stl binding sites. Figure shows relative orientations of the *stl*, *str*, and *xis* genes, and the relevant oligonucleotides (see Table A in S1 text for the sequences of the oligonucleotides) used in this study. Tick marks denote oligonucleotides to which Stl binding was experimentally observed, while in cases of oligonucleotides marked by a cross sign no specific Stl binding was detected. The important regions are also highlighted. The locations of the important segments are marked in the following pattern: (i) the promoter regions predicted by BPROM (Table C in S1 text) are underlined (*str*: dashed line, *stl*: dotted line, *xis*: line), (ii) The overlapping regions between the *str-stl*, and *str-xis* intergenic regions are labeled with boxes (red and blue), (iii) the mutated bases are marked with magenta, (iv) the motifs that were identified by MEME are highlighted by bold lettering, and (v) the bases that are part of the identified inverted repeat are labeled by capital lettering. The SaPIs—as the bacteriophages—are diagrammed with the c1 repressor at the left of the switch region. The representation of genes follows this directionality. Numbering of both strands (+ strand/—strand) is indicated. The + strand is numbered according to the SaPIbov1 GenBank: AF217235.1 sequence. We also note that the “gaattc” segment in the stl originates from the subcloning vector, as it was described before [21].

With regards the *in vitro* investigation of Stl DNA binding, recently, we have shown that the DNA binding segment of the Stl protein is located at the N-terminal part of the protein, in a predicted helix-turn-helix (HTH) motif [23]. In Electrophoretic Mobility Shift Assay (EMSA) experiments Stl was shown to bind to a 183-bp-long DNA segment that constituted a part of the *stl* and *str* intergenic region and the beginning of Stl gene (termed here as “stl”, Fig 1) [3,21].

Although these expression studies and *in vitro* investigations have revealed important details of Stl-related gene expression regulation, the specific binding site of Stl on the SaPIbov1 genome has not been identified. Our aim in this study was to pinpoint the DNA sequence element(s) that constitute a minimal binding site for Stl. With this aim, we designed a number of oligonucleotides and followed a rational pattern of motif identification, in which we have used *in silico* predictions and EMSA. Our results show that there are at least three predicted binding motifs for Stl located at different regions within SaPIbov1, and each of these binding motifs constitutes 23 base pairs.

## Materials and Methods

### Expression and purification of Stl protein

Stl expression and purification were performed as described previously [21,24]. Briefly, Stl protein was expressed from the vector pGEX-4T-1 previously described in Szabó et al [21]. This vector was transformed intro BL21 (DE3) Rosetta cells. The transformed culture was grown in LB till exponential growth phase at 37°C, then the culture was induced with 0.5 mM isopropyl-β-D-thiogalactosidase. The protein expression was prepared at 30°C for a further 4 hours. Then the cells were centrifuged and stored at -80°C.

For Stl purification, the cells were lysed and stirred on ice for 10 min in 15 ml buffer A (PBS pH 7.3, 5 mM MgCl_2_), with 400 mM NaCl, 2 mM dithiothreitol (DTT), 1% Triton X-100, 2 μg/ml RNase, DNase and one tablet of protease inhibitor (Complete ULTRA Tablets, Mini, EDTA-Free). The suspension was sonicated for 4 x 60 s and centrifuged in 16000 g for 30 min. Supernatant was diluted in buffer A (supplemented with 200 mM NaCl) and loaded into 3 ml glutathione-agarose affinity column (GE Healthcare) and then washed with ten volume of buffer A supplemented with 200 mM NaCl. An overnight thrombin cleavage was performed on column with 80 Cleavage Unit thrombin (GE Healthcare), followed by elution of Stl protein at approx. 95% purity, based on SDS-PAGE analysis. The protein preparation was frozen in 400 mM NaCl buffer in liquid nitrogen and stored in -80°C at 1-1.5 mg/ml concentration in 30 μl volume aliquots. Before use, aliquots were centrifuged and protein concentration was measured.

### Oligonucleotide hybridization

Reaction mixtures were prepared in a total volume of 20 μl consisting of 9 μl of 1 mM stock solutions of each forward and reverse oligonucleotides (synthesized by Eurofins MWG Operon) and 2 μl annealing buffer (100 mM Tris HCl, 1 M NaCl, 0.5 M EDTA pH 7.5). Reaction mixtures were thermostatted at 95°C for 5 min, followed by a rapid centrifugation step. After centrifugation, the supernatant fraction was let to cool down to room temperature in 60 minutes during which time annealing of the oligonucleotide pairs to form double-stranded DNAs has occurred. Double-stranded oligonucleotide solutions were aliquoted into 2 μl portions and stored at -80°C.

### EMSA

Oligonucleotides and Stl protein were mixed in EMSA buffer (PBS (pH 7.3), 5 mM MgCl_2_, 75 mM NaCl, 0.5 mM EDTA) in 20 μl total volume. The samples were incubated for 15 min at room temperature. 8% polyacrylamide gels were electrophoresed in Tris- Borate- EDTA (TBE) buffer at 100 V for one hour, followed by loading of the samples. Electrophoresis was then performed in TBE buffer for about 60 min at room temperature at 150 V. Gels were analyzed by a Uvi-Tec gel-documentation system (Cleaver Scientific Ltd., Rugby, UK) using GelRed staining (Biotium) [3,25].

### Motif prediction

#### MEME

DNA motif analysis was performed by the MEME (Multiple Expectation maximization for Motif Elicitation) software. This program uses an expectation-maximization algorithm to discover motifs in the data set of DNA sequences. The motif analysis was performed by the program setting the parameters to use only the input strand and allow search for palindromes [26–28].

#### Promoter prediction

For promoter prediction the BRPOM software was used (http://linux1.softberry.com/berry.phtml?topic=bprom&group=programs&subgroup=gfindb) [29]. This software is a bacterial σ 70 promoter recognition program with about 80% accuracy and specificity [30–33]. The respective genes were analyzed together with their upstream region. In case of *stl* and *str* the *stl-str* intergenic region (98 bases) plus the respective coding sequences were used for the analysis. In case of *xis*, the upstream intergenic region between *str* and *xis* is very short, therefore here the upstream 100 bases was used, together with the coding sequence of the *xis* gene. In all cases, the strands containing the coding information were analyzed.

#### BLAST search

Pairwise alignment of intergenic region between *stl-str* and *str-xis*, was done with BLAST (http://blast.ncbi.nlm.nih.gov/Blast.cgi) using the blastn algorithm. The expect threshold was set to 0.01, other parameters were not changed. The presence of additional predicted Stl binding sites within the SaPIbov1 (GenBank: AF217235.1) genome was checked also with blastn, using the default parameters. The search was automatically optimized for short sequences.

## Results

### Stl binds to the *stl-str* intergenic region

**Fig 2:**
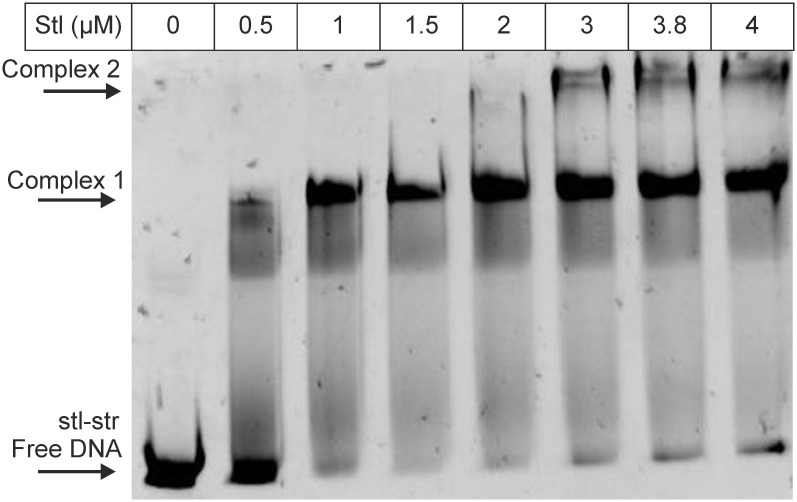
EMSA panel showing the binding of Stl to the oligonucleotide stl-str. Stl concentration is shown in the top row; arrows indicate positions of free DNA and the formed Stl-DNA complexes.

Based on the expression studies [2,22] it is expected that Stl binds to the intergenic region between the *stl* and *str* genes. However, the previously used DNA segments did not contain the whole intergenic region of these two genes [3,21], thus, we extended the originally tested oligonucleotide (“stl”, Fig 1, and Table A in S1 text, note that on Fig 1 the “-”strand of SaPIbov1 is presented, and the text refers also to the “-”strand in every case) [21] to contain the whole intergenic region (termed “stl-str”, Fig 1, Table A in S1 text). We performed EMSA experiment with this oligonucleotide, and found that Stl binds to this segment (Fig 2). The EMSA gel indicates also the putative presence of two different complexes (Complex 1 and Complex 2 on Fig 2) formed between Stl protein and the stl-str oligonucleotide. This may suggest the potential existence of two Stl binding sites within the investigated DNA segment.

### Stl binds to the *str-xis* intergenic region

**Fig 3:**
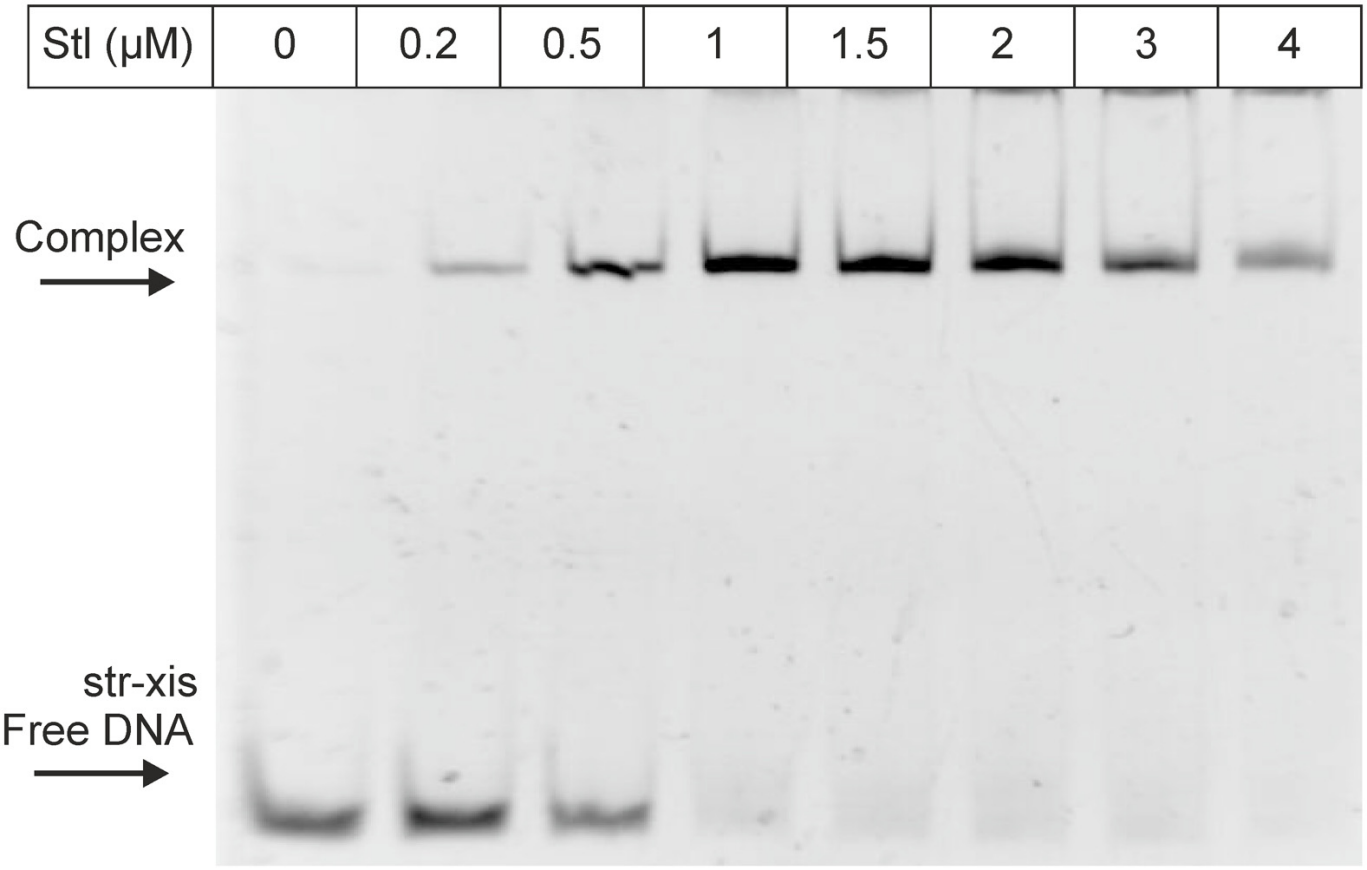
EMSA panel showing the binding of Stl to the oligonucleotide str-xis. Stl concentration is shown in the top row; arrows indicate positions of free and complexed DNA.

Previously it was found, that Stl represses the *xis* gene [2,22]. Therefore, we tested whether Stl binds to the upstream region of the *xis* gene, using the str-xis oligonucleotide (Fig 1, Table A in S1 text), which contained the whole *str-xis* intergenic region. We found that, indeed, Stl binds to this DNA segment (Fig 3).

### Rational design of oligonucleotides for binding tests using an *in silico* approach

In order to pin down a shorter DNA segment that allows Stl binding, we designed shorter oligonucleotides and performed EMSA tests. For the rational design of these oligonucleotides we used an *in silico* prediction-based approach, as detailed below.

Stl was suggested to activate its own expression [2] proposing that it may bind to the non-coding region between the *stl* and *str* genes, and not within the coding region of Stl. Besides, expression of Str and Xis proteins are both repressed by Stl, therefore we expected that the Stl binding sites at these two regions may be similar. Thus, to predict the exact binding sites, we analyzed the *stl-str* and the *str-xis* intergenic regions. First, we aligned the sequences of these regions with nBLAST algorithm. As a result, we got two significant matches (Table B in S1 text). These hits were overlapping in the str-xis sequence, but they were separated in the stl-str sequence (Fig 1, marked with red and blue boxes).

In addition, we also performed a promoter prediction with the BPROM software [29–33] (Table C in S1 text). As based on the expression pattern it may be expected that the Stl binding site overlaps with the promoters of the regulated genes. The predicted possible -10 and -35 regions are shown on Fig 1, indicated by dashed underlining.

We have also subjected the stl-str and str-xis oligonucleotides to the MEME motif finder tool [26]. However, at this stage of our studies, the MEME analysis did not result in any significant hits.

**Fig 4:**
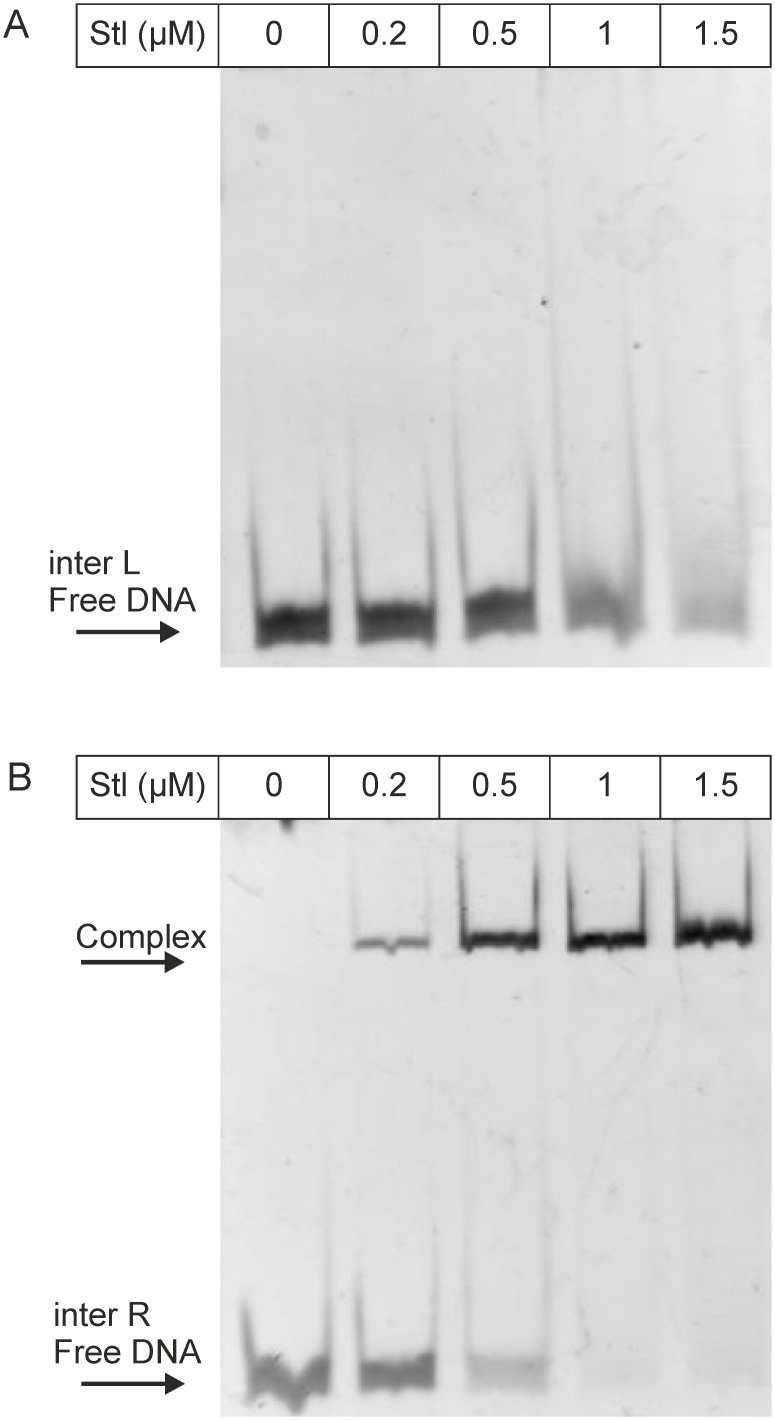
EMSA panel showing the binding of Stl to the oligonucleotide inter-L (A) and inter-R (B), respectively. Stl concentration is shown in the top row; arrows indicate positions of free and complexed DNA.

Based on the results of the BLAST alignment and the promoter prediction, we have split the *stl-str* intergenic region into two parts of equal lengths (inter-R and inter-L, where R and L correspond to the segments proximal to the *str* and *stl* genes, respectively, throughout this text), so that all of the predicted potential binding sites were involved (Fig 1, Table A in S1 text). The results of the EMSA test shown on Fig 4 indicates that Stl binds specifically to the sequence inter-R. In the case of oligonucleotide inter-L unlikely of the other oligonucleotides examined before no significant binding was observed at 1.5 μM Stl concentration. Based on these results first we investigated further the inter-R segment, which was cut again into two equal parts, so that all predicted potential binding sites were included (inter-RR and inter-RL, Fig 1, Table A in S1 text). Neither of these two segments showed any binding (S1 Fig). Taking together these data suggested that the binding motif may be located in the middle part of inter-R, and the cleavage of the sequence inter-R into two halves may have destroyed the binding site.

**Fig 5:**
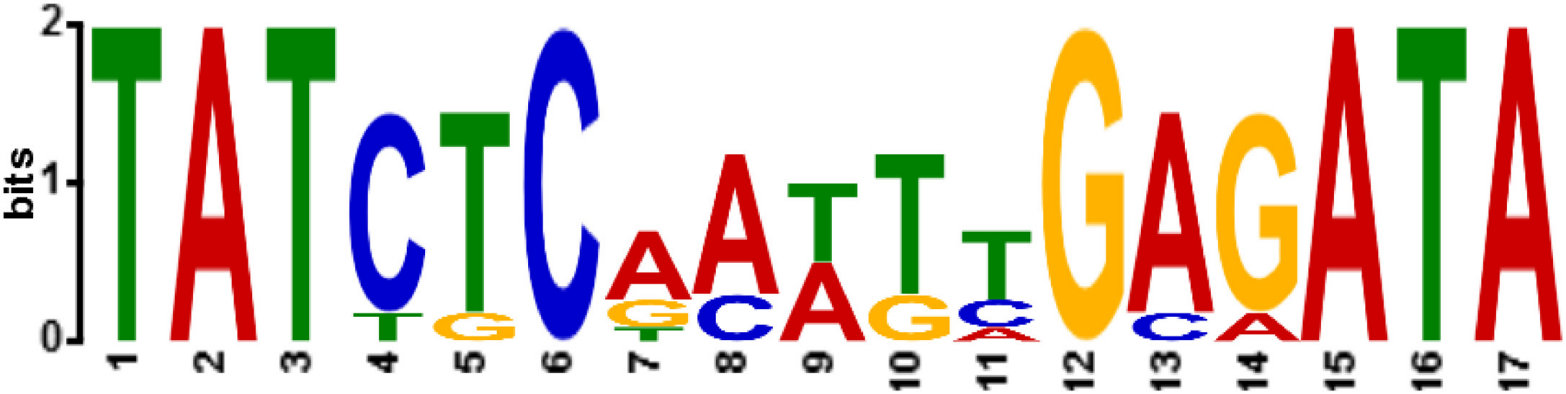
Logo representation of the identified Stl binding motif. The logo representation was generated from the alignment of stl, stl-str, str-xis and inter-R, using the MEME predictor [27]. Height and size of letters at any positions represent the probability of each possible base appearing at the given position within the predicted motif. We searched for palindromes, which causes MEME to average the letter frequencies in corresponding motif columns together. Thus, if the width of the motif is 10, columns 1 and 10, 2 and 9, 3 and 8, etc., are averaged together. The averaging combines the frequency of A in one column with T in the other, and the frequency of C in one column with G in the other.

At this point, to identify the predicted binding motifs we turned back to the MEME motif finder tool. This time, we used all of the oligonucleotides that were capable of binding to Stl, i.e. the sequence input included sequences stl, stl-str, str-xis as well as inter-R. With this increased input, the MEME software led to the identification of a 17-mer motif, containing an inverted repeat (Fig 5). We found two variants of this motif in the *stl-str* intergenic region and one in the *str-xis* intergenic region. These variants were found to be located upstream from either the *str*, the *stl* or the *xis* genes, therefore we termed these motif variants as Motif_str_, Motif_stl_, and Motif_xis_ (Fig 1, bold), respectively. Importantly, these motifs are overlapping with the predicted promoter regions of the respective proteins (Fig 1, underlined regions).

In Motif_str_ and Motif_xis_, the inverted repeat region is identical and symmetric, while in Motif_stl_ two bases at the 3’ side of the inverted repeat are altered as compared to Motif_str_ and Motif_xis_. These alterations destroy the inverted repeat at this site. Still, Motif_stl_ is the single motif that could be identified in our present studies located within the sequence stl, which was originally described as the binding site of the Stl protein [3].

Interestingly, Motif_stl_ was present in inter-L, although this oligonucleotide did not show specific binding (Figs 1 and 4). However, the 17-mer predicted sequence was very close to the 3’end of the inter-L oligonucleotide, with only one single overhanging nucleotide. We hypothesized that Stl needs some extra, nonspecific overhanging segment for the binding. We set out to test the predicted binding motifs and the necessity of the overhang.

### Verification of the predicted Stl binding sites in *in vitro* experiments

**Fig 6:**
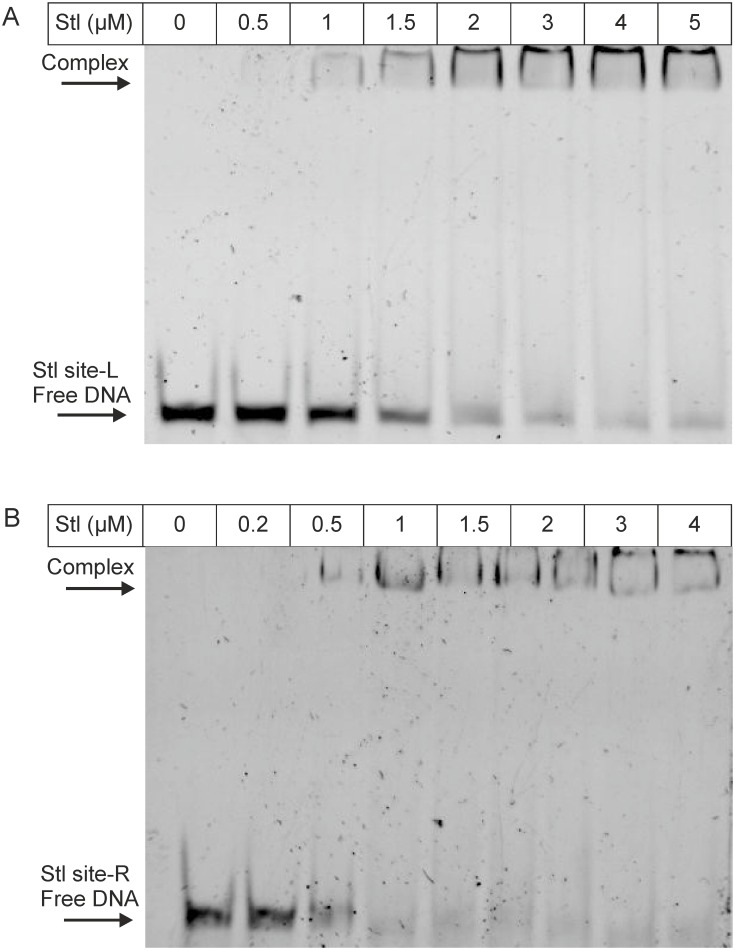
EMSA panel showing the binding of Stl to the oligonucleotide Stl site-L (A) and Stl site-R (B), respectively. Stl concentration is shown at the top row; arrows indicate positions of free and complexed DNA.

Considering the sequence logo resulted by MEME analysis, we expected that the inverted repeat present in the 17-mer is of high importance in the specificity in Stl binding. As the inverted repeat region was found to be identical in Motif_str_ and Motif_xis_, and it differed in Motif_stl_, we have chosen Motif_str_ and Motif_stl_ for the verification of the predictions. The motifs were also extended both 3’ and 5’ direction with 3 base pairs, to provide overhangs required for the binding capability of the repressor, resulting in the test oligonucleotides (Stl site-R and Stl site-L, Fig 1). Both Stl site-R and Stl site-L clearly showed binding to the Stl protein (Fig 6), indicating that the motifs were predicted correctly.

To verify the importance of the overhang, the inter L+tt and the Stl site-L–tt oligonucleotides were designed. The inter L+tt contained two extra thymines at the 3’ end compared to inter L, while Stl site-L-tt lacked two thymines in the same position compared to Stl-site-L (Fig 1). Inter L+tt showed binding capability, unlike the Stl site-L-tt (S2 Fig), proving that the overhang is needed for Stl to bind its’ specific binding site.

To investigate the importance of the inverted repeat region in the binding, we prepared a variant of Stl site-R, which contained adenines (polyA) between the two sides of the inverted repeat and also at both ends, as a non-specific spacer/overhang. This oligonucleotide was termed as Stl site-R polyA. This modification did not disturb the binding capability of Stl (S3 Fig Panel A), confirming that the nucleotides originally present at these spacer and overhang locations do not contribute to Stl binding, and they can be replaced by the polyA tracts.

To directly test if the palindrome region has a high importance in the Stl-DNA complex formation, we generated two point mutations in Stl site-R polyA (Stl site-R G17T and Stl site-R C7G). These mutations significantly weaken the palindromic character of the oligonucleotides in question, since even the purine-purine or pyrimidine-pyrimidine symmetry has not been preserved at that specific position. We found that these mutations led to a highly attenuated binding, equivalent to that observed with an aspecific oligonucleotide (S3 Fig Panels B and C to be compared with S4 Fig). These data strongly support our hypothesis that the palindromic character of the oligonucleotide plays an important role in the formation of the Stl-DNA complex.

**Fig 7:**
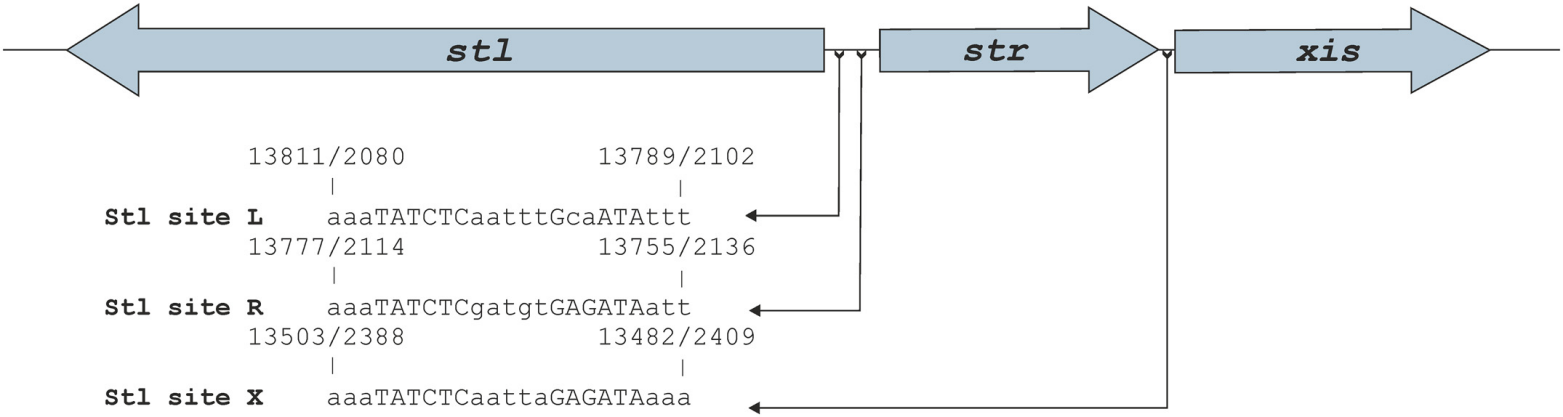
Schematic representation of the Stl binding sites and their relative locations compared to the affected genes. The sequences and the relative locations of the three identified Stl binding sites are shown. The bases that are part of the inverted repeat are highlighted by capital lettering.

In summary, we have shown that the Stl binding motif consists of a 6-bp inverted repeat separated by a 5-bp nonspecific spacer sequence (Fig 7). Stl also requires at least 3-bp overhangs for binding, thus, the resulting Stl binding site is 23-bp. With regard to the sequences of the identified binding sites, two out of the three, Motif_str_ and Motif_xis_ contain a perfectly symmetric inverted repeat, while in the third binding site, Motif_stl_, the symmetry is broken by the alteration of two bases (Fig 7) at the 5’ side of the repeat, indicating that some specific mutations of the inverted repeat may be tolerated (Stl site-L). The defined 23 base pairs long Stl predicted binding sites are located upstream from *stl*, *str* and *xis* genes, and importantly these predicted binding sites are overlapping also with the predicted promoter regions of the respective genes. No further potential binding sites with similar sequences were identified within the DNA sequence of SaPIbov1 using blastn analysis.

## Discussion

There are several well characterized gene regulatory proteins such as *lac* [34], *trp* [35] and λ phage CI repressors [36], which are known to utilize inverted repeat or palindromic DNA sequences as binding sites for the relevant repressor protein molecules, similarly to the system investigated in this study. In the case of the *E*. *coli* arginine repressor, the repressor-operator site contains a 18-bp inverted repeat sequence termed as ARG boxes [37]. Similarly, the *S*. *cerevisiae* transcription factor ADR1 regulates ADH2 expression through a 22-bp inverted repeat sequence (UAS1) [38]. The vast majority of prokaryotic transcriptional regulatory proteins contain domains with helix-turn-helix (HTH) motif for specific DNA binding [39–41]. This structural motif also is present in the Stl protein [23]. Moreover, HTH proteins usually bind as dimers onto the DNA. In this respect, it might be worthwhile to point out that Stl was also suggested to be capable of dimerization [21]. It is possible that Stl binds to the inverted repeat region as a dimer, and the HTH motif of one subunit interacts with one side of the repeat, while the HTH motif of the other subunit interacts with the other side of the repeat, as for example in the case of the λ phage CI repressor [39,42]

In addition to the determination of the sequences of the specific Stl binding sites, we have also investigated the relative locations of the predicted Stl binding sites compared to the locations of the *stl*, *str* and *xis* genes and to the predicted promoter regions of the respective genes. Two of the three predicted Stl binding sites are located within the *str-stl* intergenic region, while the *str-xis* intergenic region also contains one of those (Fig 7). These are also corroborated by the experimental finding that there are two different DNA complexes formed between Stl and the DNA probe containing the whole *stl-str* intergenic region (Fig 1) present in the EMSA gel, while Stl forms one kind of complex with the oligonucleotide containing the *str-xis* region (Fig 2). Furthermore, we have found that Stl site-R and Stl site-X overlap the -10 regions of the predicted promoter regions of *str* and *xis*, respectively (Fig 1). This provides a straightforward explanation for the repression of *str* and *xis* by Stl, namely we propose that Stl binding to these segments within the predicted promoter region inhibits the formation of the transcription initiation complex at these sites.

We have also shown that the Stl site-L overlaps with the predicted -35 box of the *stl* gene (Fig 1). How this results in the possible autoinduction of Stl [2] is less obvious, than the repression mechanism of *str* and *xis* genes. However, there are some examples, where transcription factor binding to the -35 promoter box activates the expression of the respective genes (Class II activation [43] e.g. λ phage CI repressor [44]), or between the -10 and -35 regions (Class III activation [43], such as the MerR type activators [43,45]. Transcriptional activators that bind to the -35 box interact with certain region (region 4) of the sigma factor, and aid the transcription initiation by the recruitment of RNA polymerase–sigma factor complex. The best example for this mechanism is the CI protein of lambda phage that is responsible for the repression of λ phage lytic cycle activation. Transcriptional activation may be the result also of activator binding between -35 and -10 box. In this case, the activator binding alters the conformation of the promoter and reorients the -10 and -35 elements so that the binding ability of RNAP-sigma factor complex to the promoter is improved [43]. The Stl site-L overlaps with the predicted -35 box, and it expands also the spacer region between the possible-35 and -10 promoter boxes. Therefore, it is possible that Stl autoactivates its own expression by either Class II or Class III mechanisms. It is also important to highlight that this predicted Stl binding site is the one which has a broken symmetry due to the base alterations of one side of the inverted repeat. This may have an impact also on the function and it may also be relevant for the activation.

Importantly, transcriptional activators may also bind upstream from promoters. These activators act by interacting with the C-terminal domain of the RNA polymerase α subunit (Class I activation [43]), such as in the case of the cyclic AMP receptor protein (CRP) which is the activator of the lac promoter. The predicted Stl binding site-R that overlaps also with the possible -10 box of *str* is in similar position compared to the *stl* promoter (Fig 1 and S5 Fig) as the CRP activator compared to the *lac* promoter. Therefore, we cannot rule out that Stl binding to this site may also contribute to Stl activation. In fact, examples for complex activation processes have also been presented in the literature, where multiple types of the above mentioned mechanisms are present simultaneously [43]. Nevertheless, the observed autoactivation of Stl expression may also be related to other transcription factors of the SaPIbov1.

With regard to the *int* gene, earlier studies showed that Stl represses expression from this gene, however, we could not identify any other Stl binding sites directly upstream of *int*. The lack of an additional Stl binding site suggests that this gene may be controlled by Stl binding to the above described three binding sites, however, the exact mechanism of Stl controlled *int* regulation remains elusive.

In conclusion, by the determination of predicted Stl binding sites using *in vitro* experiments, together with the *in silico* analysis of the possible promoter regions of the affected genes, our study sheds light on the possibility of several regulation mechanisms and offers a framework for *in vivo* studies on these systems of high biomedical interest.

To further verify the predicted Stl binding sites DNase I footprinting studies are to be done. Consequent *in vivo* validation of the assignment of the binding sites and assessment of their significance in excision and replication of SaPIbov1 is to be performed as a continuation of this work.

## Supporting Information

S1 Fig EMSA panels (A and B) of inter RR and inter RL, respectively. Stl concentration is shown in the top row; arrow indicates position of free DNA.
(TIF)

S2 Fig EMSA panels (A and B) of inter-L+tt and Stl site-tt, respectively. Stl concentration is shown in the top row; arrow indicates position of free DNA.
(TIF)

S3 Fig EMSA panels (A, B and C) of Stl site-R poliA, Stl site-R G17T and Stl site-R C7G, respectively. Stl concentration is shown in the top row; arrow indicates position of free DNA.
(TIF)

S4 Fig EMSA panel of seq aspecific. Stl concentration is shown in the top row; arrow indicates position of free DNA.
(TIF)

S5 Fig Schematic representation of the Stl binding sites and their relative locations of -10 and -35 boxes of the affected genes. The repressor or activator effects of the Stl protein exerted on to the respective genes are also indicated with blunt ended and arrow-headed lines, respectively.
(TIF)

S1 Text This document includes Supplementary Tables. Supplementary Table A lists the sequences of all oligonucleotides used in this study. Supplementary Table B presents the summary of the pairwase alignment of the stl-str and the str-xis intergenic regions, while Supplementary Table C provides a summary of the BPROM promoter prediction for *stl*, *str* and *xis* genes. The oligonucleotide labelled as ‘aspecific’ was selected as a 60 bp long segment of the *S aureus* genome (14170 -14230). This segment is devoid of any identifiable motif and shows no similarity to the stl-str or str-xis. Figure S1 shows EMSA results using the aspecific oligonucleotide and indicates that there is a low degree of binding, however, this binding is significantly less strong as compared to binding of stl-str (cf main text Figure 2). We termed this binding pattern as “aspecific binding”. We observed that this aspecific binding pattern is present with the inter RR, inter RL, Stl site-R C7A and Stl site-R G17C oligonucleotides as well (Supplemental Figures 2 A and B, 3 B and C). The Stl site-R polyA oligonucleotide on the other hand (cf Supplemental Figure 3 A) bound to the Stl protein with comparable affinity to the Stl site-R and Stl site-L oligonucleotides (cf main text Figure 6 A and B).
(DOCX)
