# Supplementary figures and images for: In Vitro Analysis of Predicted DNA-Binding Sites for the Stl Repressor of the Staphylococcus aureus SaPIBov1 Pathogenicity Island
Source: PLoS One. 2016 Jul 7;11(7):e0158793. doi: 10.1371/journal.pone.0158793 (PMC4936726; doi:10.1371/journal.pone.0158793)

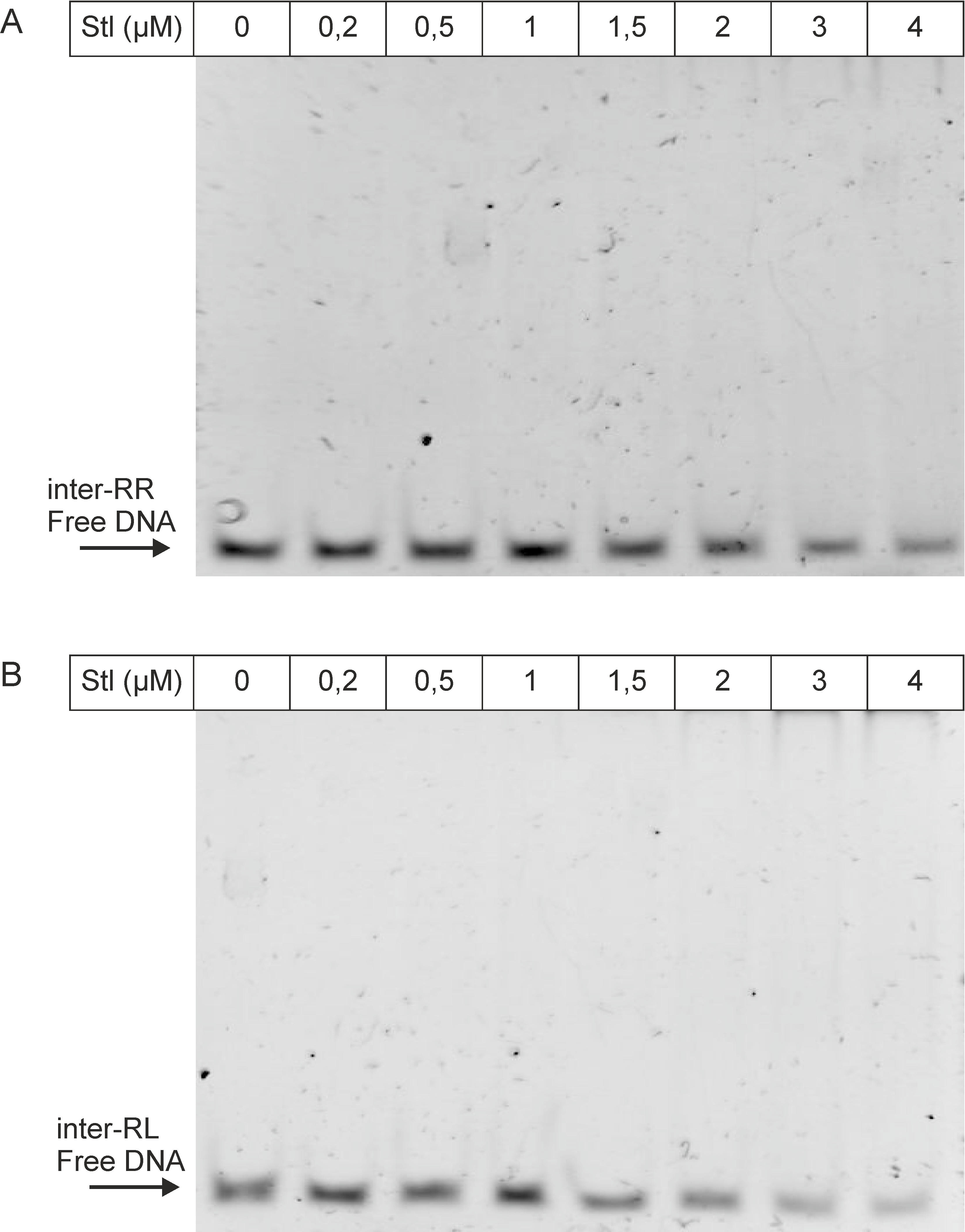

Supplement: S1 Fig — Stl concentration is shown in the top row; arrow indicates position of free DNA. (TIF) [file pone.0158793.s001.tif]

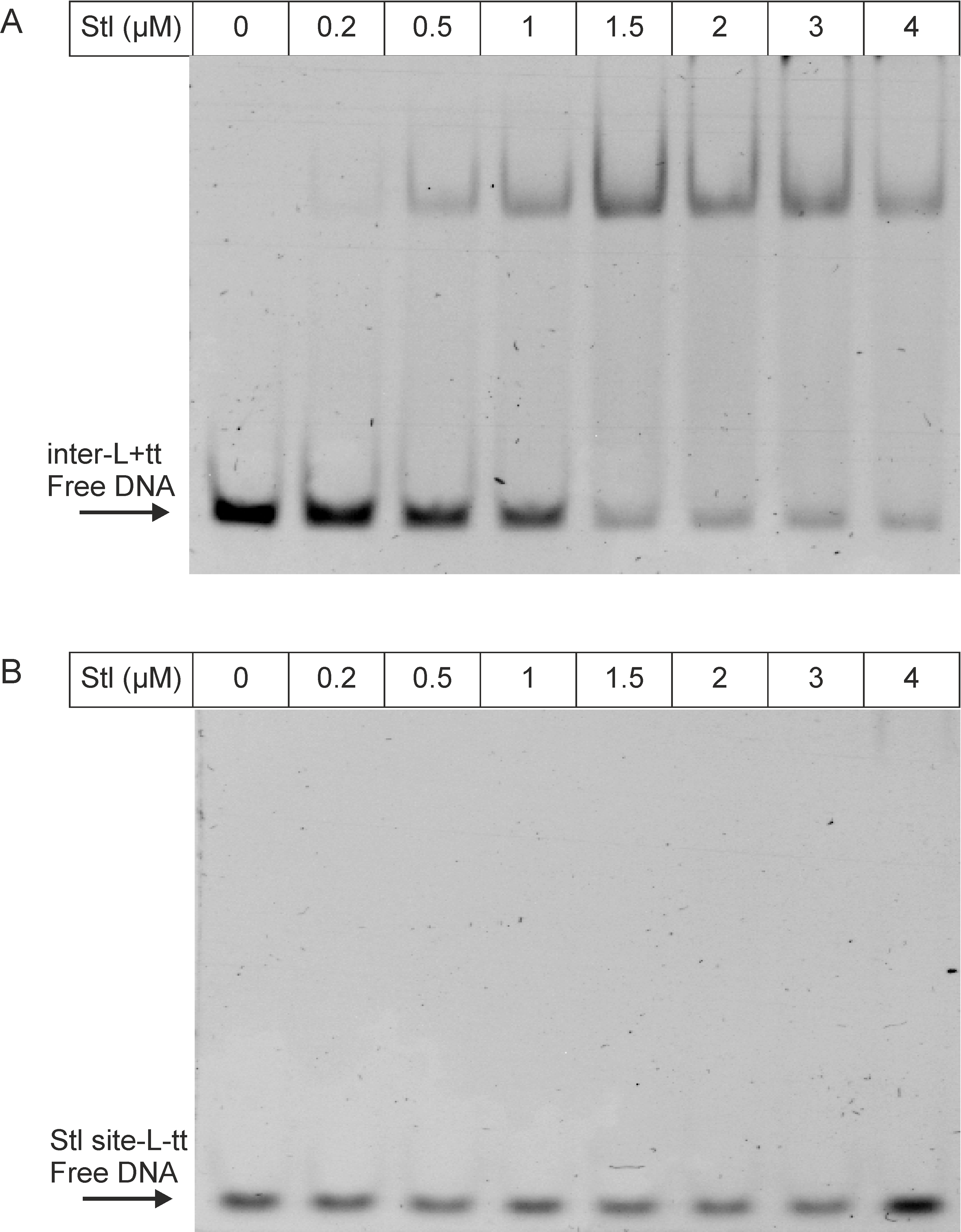

Supplement: S2 Fig — Stl concentration is shown in the top row; arrow indicates position of free DNA. (TIF) [file pone.0158793.s002.tif]

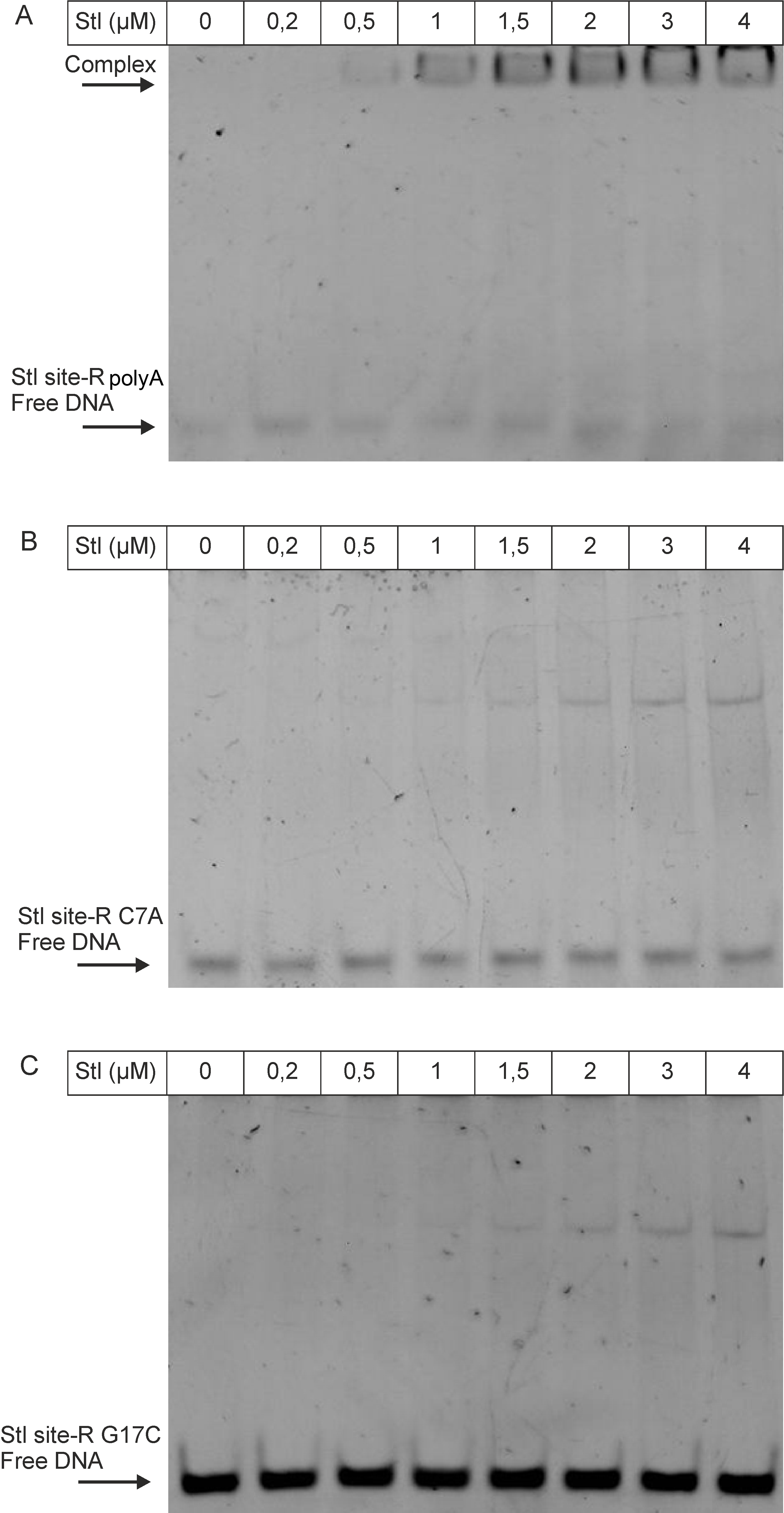

Supplement: S3 Fig — Stl concentration is shown in the top row; arrow indicates position of free DNA. (TIF) [file pone.0158793.s003.tif]

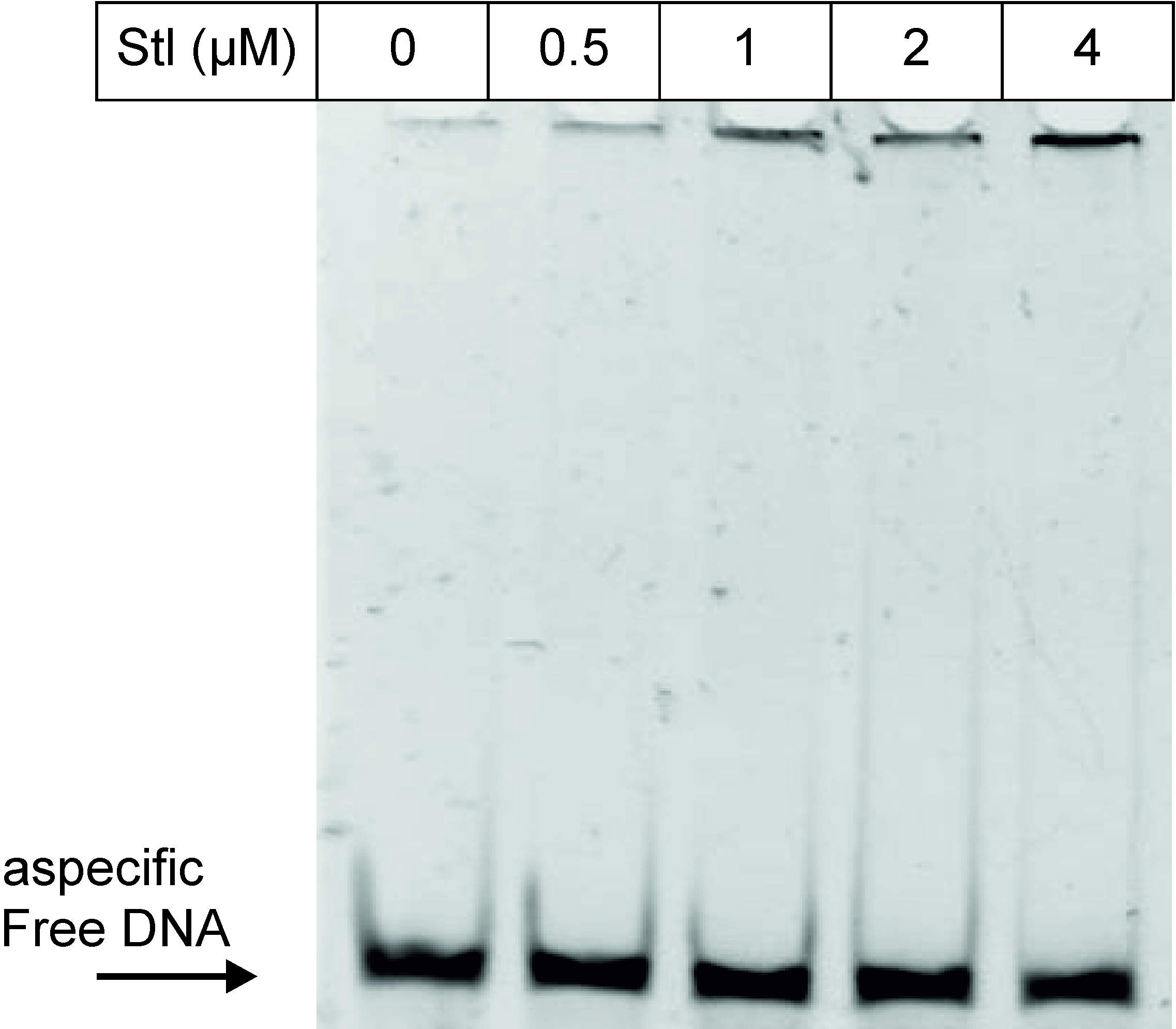

Supplement: S4 Fig — Stl concentration is shown in the top row; arrow indicates position of free DNA. (TIF) [file pone.0158793.s004.tif]

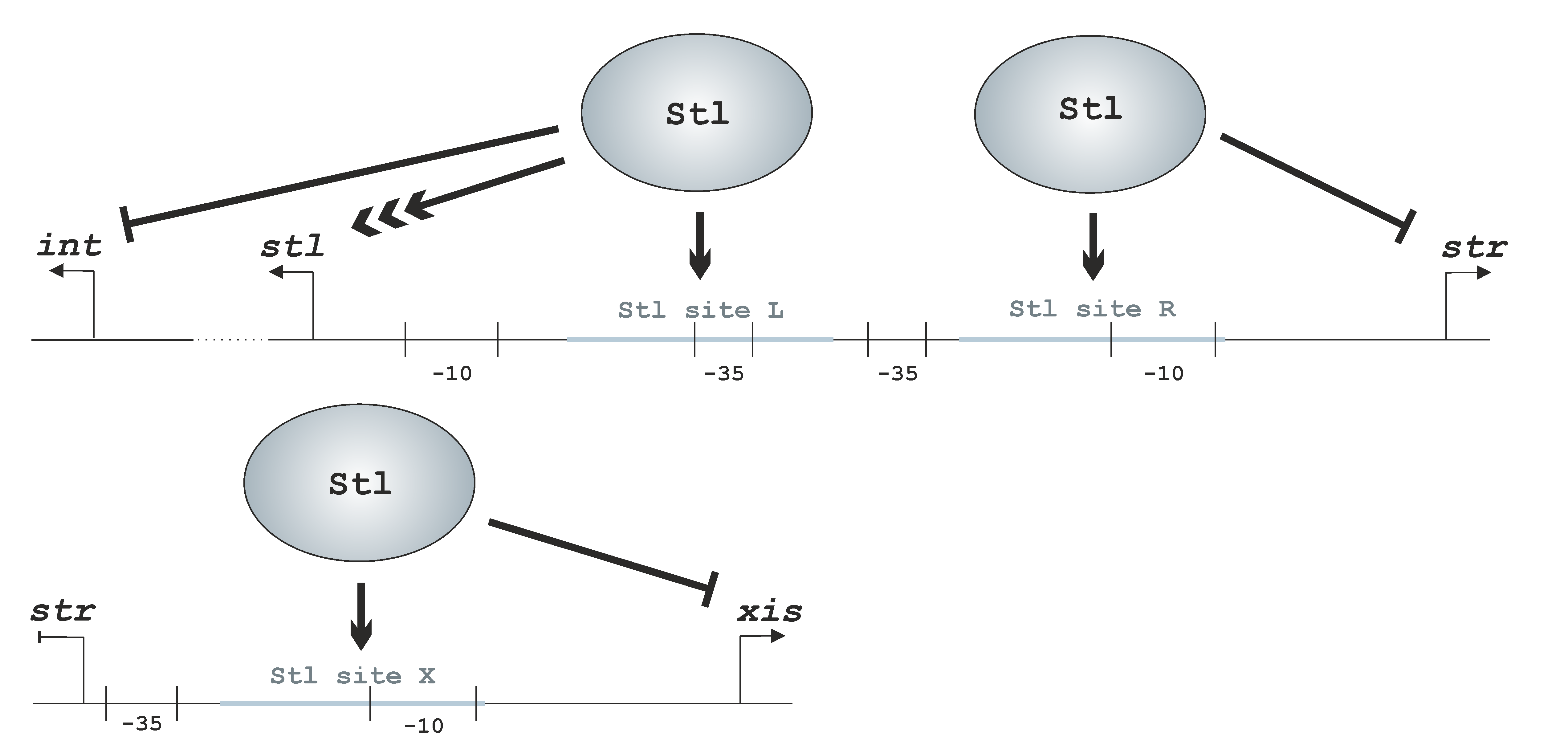

Supplement: S5 Fig — The repressor or activator effects of the Stl protein exerted on to the respective genes are also indicated with blunt ended and arrow-headed lines, respectively. (TIF) [file pone.0158793.s005.tif]
