# Supplementary material for: In Vitro Analysis of Predicted DNA-Binding Sites for the Stl Repressor of the Staphylococcus aureus SaPIBov1 Pathogenicity Island
Source: PLoS One. 2016 Jul 7;11(7):e0158793. doi: 10.1371/journal.pone.0158793 (PMC4936726; doi:10.1371/journal.pone.0158793)
Supplement: S1 Text — Supplementary Table A lists the sequences of all oligonucleotides used in this study. Supplementary Table B presents the summary of the pairwase alignment of the stl-str and the str-xis intergenic regions, while Supplementary Table C provides a summary of the BPROM promoter prediction for stl, str and xis genes. The oligonucleotide labelled as ‘aspecific’ was selected as a 60 bp long segment of the S aureus genome (14170 -14230). This segment is devoid of any identifiable motif and shows no similarity to the stl-str or str-xis. Figure S1 shows EMSA results using the aspecific oligonucleotide and indicates that there is a low degree of binding, however, this binding is significantly less strong as compared to binding of stl-str (cf main text Figure 2). We termed this binding pattern as “aspecific binding”. We observed that this aspecific binding pattern is present with the inter RR, inter RL, Stl site-R C7A and Stl site-R G17C oligonucleotides as well (Supplemental Figures 2 A and B, 3 B and C). The Stl site-R polyA oligonucleotide on the other hand (cf Supplemental Figure 3 A) bound to the Stl protein with comparable affinity to the Stl site-R and Stl site-L oligonucleotides (cf main text Figure 6 A and B). (DOCX) [file pone.0158793.s006.docx]

**Supporting Information**

for

***In vitro* analysis of predicted DNA-binding sites for the Stl repressor of the *Staphylococcus aureus* SaPIBov1 pathogenicity island**

Veronika Papp-Kádár^1,2,^^[[1]](#footnote-1)^ , Judit Eszter Szabó^1,2,i^, Kinga Nyíri^1,2^, Beata G Vertessy^1,2,*^

^1^ Institute of Enzymology, Research Centre for Natural Sciences, Hungarian Academy of Sciences, Budapest, 1117, Hungary

^2^ Department of Applied Biotechnology and Food Science, Budapest University of Technology and Economics, Budapest, 1111, Hungary

* To whom correspondence should be addressed. Beata G Vertessy, Tel: +36 1 382 6707; Email: vertessy.beata@mail.bme.hu;

Veronika Papp-Kádár, Tel: +36 1 382 6729; Email: kadar.veronika@ttk.mta.hu

Supplementary Table I lists the sequences of all oligonucleotides used in this study. Supplementary Table II presents the summary of the pairwase alignment of the stl-str and the str-xis intergenic regions, while Supplementary Table III provides a summary of the BPROM promoter prediction for *stl*, *str* and *xis* genes.

The oligonucleotide labelled as ‘aspecific’ was selected as a 60 bp long segment of the *S aureus* genome (14170 -14230). This segment is devoid of any identifiable motif and shows no similarity to the stl-str or str-xis. Figure S1 shows EMSA results using the aspecific oligonucleotide and indicates that there is a low degree of binding, however, this binding is significantly less strong as compared to binding of stl-str (cf main text Figure 2). We termed this binding pattern as „aspecific binding”.

We observed that this aspecific binding pattern is present with the inter RR, inter RL, Stl site-R C7A and Stl site-R G17C oligonucleotides as well (Supplemental Figures 2 A and B, 3 B and C). The Stl site-R polyA oligonucleotide on the other hand (cf Supplemental Figure 3 A) bound to the Stl protein with comparable affinity to the Stl site-R and Stl site-L oligonucleotides (cf main text Figure 6 A and B).

**Table I**

**List of the oligonucleotide sequences used in this study for the EMSA experiments.**

|  | **name** | **length (bases)** | **strand** | **sequence (5'-3')** |
| --- | --- | --- | --- | --- |
| 1 | stl | 183 | + | gaattcatttcaacattaaatattgcaaattgagatatttttttcgatatgatatcatttggatggaaggagctggtcaaatggcagaattaccaacacattacggcacaattattaaaactcttagaaaatacatgaaattaactcaaagcaaattgagtgaaaggacaggatttaggatcc |
|  |  |  | - | ggatcctaaatcctgtcctttcactcaatttgctttgagttaatttcatgtattttctaagagttttaataattgtgccgtaatgtgttggtaattctgccatttgaccagctccttccatccaaatgatatcatatcgaaaaaaatatctcaatttgcaatatttaatgttgaaatgaattc |
| 2 | stl-str | 230 | + | tcgtaaacatattctcacctcctcgaacaaattatctcacatcgagatatttatttcaacattaaatattgcaaattgagatatttttttcgatatgatatcatttggatggaaggagctggtcaaatggcagaattaccaacacattacggcacaattattaaaactcttagaaaatacatgaaattaactcaaagcaaattgagtgaaaggacaggatttagtcaaaa |
|  |  |  | - | ttttgactaaatcctgtcctttcactcaatttgctttgagttaatttcatgtattttctaagagttttaataattgtgccgtaatgtgttggtaattctgccatttgaccagctccttccatccaaatgatatcatatcgaaaaaaatatctcaatttgcaatatttaatgttgaaataaatatctcgatgtgagataatttgttcgaggaggtgagaatatgtttacga |
| 3 | inter-L | 43 | + | atattgcaaattgagatatttttttcgatatgatatcatttgg |
|  |  |  | - | ccaaatgatatcatatcgaaaaaaatatctcaatttgcaatat |
|  | inter-L+tt | 45 | + | aaatattgcaaattgagatatttttttcgatatgatatcatttgg |
|  |  |  | - | ccaaatgatatcatatcgaaaaaaatatctcaatttgcaatattt |
| 4 | inter-R | 43 | + | tcctcgaacaaattatctcacatcgagatatttatttcaacat |
|  |  |  | - | atgttgaaataaatatctcgatgtgagataatttgttcgagga |
| 5 | aspecifc | 60 | + | attcatcatacgatttatatgatgaaacaatcaaattgttggaattgttgaaagaatcta |
|  |  |  | - | tagattctttcaacaattccaacaatttgattgtttcatcatataaatcgtatgatgaat |
| 6 | inter-RR | 21 | + | tcctcgaacaaattatctcac |
|  |  |  | - | gtgagataatttgttcgagga |
| 7 | inter-RL | 22 | + | atcgagatatttatttcaacat |
|  |  |  | - | atgttgaaataaatatctcgat |
| 8 | Stl site-L | 23 | + | aaatattgcaaattgagatattt |
|  |  |  | - | aaatatctcaatttgcaatattt |
| 8 | Stl site-L-tt | 21 | + | atattgcaaattgagatattt |
|  |  |  | - | aaatatctcaatttgcaatat |
| 9 | Stl site-R | 23 | + | aattatctcacatcgagatattt |
|  |  |  | - | aaatatctcgatgtgagataatt |
| 10 | Stl site-R polyA | 23 | + | ttttatctctttttgagatattt |
|  |  |  | - | aaatatctcaaaaagagataaaa |
| 11 | Stl site-R G17T | 23 | + | ttttatatctttttgagatattt |
|  |  |  | - | aaatatctcaaaaagatataaaa |
| 12 | Stl site-R C7G | 23 | + | ttttatctctttttgacatattt |
|  |  |  | - | aaatatgtcaaaaagagataaaa |
| 13 | str-xis | 57 | + | gctcatattattcctctcctaccattttatctctaattgagatatttatattcagat |
|  |  |  | - | atctgaatataaatatctcaattagagataaaatggtaggagaggaataatatgagc |

**Table II**

**Summary of the pairwaise alignment of the stl-str and the str-xis intergenic regions**

| Match | Except value | length | str-xis | | stl-str | | sequence |
| --- | --- | --- | --- | --- | --- | --- | --- |
|  |  |  | strand | position | strand | position |  |
| 1 | 5 * 10^-5^ | 13 | + | 31-43 | + | 64-76 | AATTGAGATATTT |
| 2 | 7 * 10^-4^ | 11 | + | 35-45 | + | 34-44 | GAGATATTTAT |
| 3 | 0.18 | 7 | + | 34-40 | - | 29-23 | TGAGATA |
| 4 | 0.18 | 7 | + | 38-44 | - | 59-53 | ATATTTA |

**Table III**

**Summary of the BPROM promoter prediction for *stl,* *str* and *xis* genes.**

| **Investigated gene** | **strand** | **LDF** | **-10 box** | **score** | **-35 box** | **score** | **Coding region** |
| --- | --- | --- | --- | --- | --- | --- | --- |
| ***stl*** | + | 6.86 | atcatatcg | 62 | ttgcaa | 55 | no |
|  | + | 5.56 | tattataat | 79 | atgtat | 12 | yes |
|  | + | 4.51 | aattatact | 64 | ttaaag | 31 |  |
| ***str*** | - | 5.02 | tgagataat | 46 | ttgaaa | 60 | no |
| ***xis*** | - | 5.33 | agataaaat | 70 | ctgaat | 20 |  |

1. Authors contributed equally to this study as joint first authors. [↑](#footnote-ref-1)
